# Prior Surface Integrity Assessment of Coated and Uncoated Carbide Inserts Using Atomic Force Microscopy

**DOI:** 10.3390/ma4040633

**Published:** 2011-04-06

**Authors:** Samy Oraby, Ayman Alaskari, Abdulla Almazrouee

**Affiliations:** Department of Mechanical Production Technology, College of Technological Studies, PAAET, P.O. Box 42325, Shuwaikh 70654, Kuwait

**Keywords:** AFM contact mode, carbide inserts, coating surface defects and flaws, AFM surface “Roughness” and “Section”

## Abstract

Coated carbide inserts are considered vital components in machining processes and advanced functional surface integrity of inserts and their coating are decisive factors for tool life. Atomic Force Microscopy (AFM) implementation has gained acceptance over a wide spectrum of research and science applications. When used in a proper systematic manner, the AFM features can be a valuable tool for assessment of tool surface integrity. The aim of this paper is to assess the integrity of coated and uncoated carbide inserts using AFM analytical parameters. Surface morphology of as-received coated and uncoated carbide inserts is examined, analyzed, and characterized through the determination of the appropriate scanning setting, the suitable data type imaging techniques and the most representative data analysis parameters using the MultiMode AFM microscope in contact mode. The results indicate that it is preferable to start with a wider scan size in order to get more accurate interpretation of surface topography. Results are found credible to support the idea that AFM can be used efficiently in detecting flaws and defects of coated and uncoated carbide inserts using specific features such as “Roughness” and “Section” parameters. A recommended strategy is provided for surface examination procedures of cutting inserts using various AFM controlling parameters.

## 1. Introduction

The cutting edge is a critical component in machining system elements: tool, workpiece and machine tool. Variability in tool wear and tool life is one of the unresolved nuisance obstacles to achieve a full optimization of the machining process. Among many other reasons [1], the manufacturing defects on the surface of the inserts can be a major source of tool wear and life variability. Wear and life variability may lead to disastrous consequences especially in automated and adaptive control machining systems [2] where the machinability information provided by the manufacturer is usually taken for granted. A pre-examination of the inserts is, therefore, a beneficial strategy especially when the amount of time and money consumed are justified. On an economical and feasibility justified basis, this can be carried out either within the manufacturer quality control or in the research labs’ procedures.

The integrated coated surface system usually consists of the substrate, the interface and the coating layer(s). Each of these components affects, individually and interactively, the performance of the surface system under practical operating circumstances. Mono- and multi-layer coated carbide inserts have recently gained wide acceptance for use in machining of steel. Applying a thin coating layer(s) of carbides, nitrides, ceramic alloys, cermets, or metastable materials such as diamond and cubic boron nitride to the original material usually improves wear rate with less frequent catastrophic failure especially if it is used in hostile environments of high heat and friction. In general, coated carbide inserts are recommended whenever longer tool lives, better finish and higher productivity are required. Thin coating layers are conventionally deposited by various processes such as chemical vapor deposition (CVD) [3,4], physical vapor deposition (PVD) [5,6], medium-temperature CVD and plasma-activated CVD [4,7].

Defects of the coated inserts usually degrade the toughness of the coating layer(s) and thereby may lead to partial or gross coating failures. One of the common defects in PVD coating is the macro-particles and craters that can be classified into: pinholes or craters, droplets, and partly covered droplets [8]. Defects in these forms are due to droplets incorporated during film growth and the pinholes are generated as a result of debonding of macro-particles from the coating [9]. At the manufacturing stage, the droplet problem can be dealt with using some techniques such as the distributed discharge arc, steered arc or arc with magnetic field filter [9].

**Figure 1:**
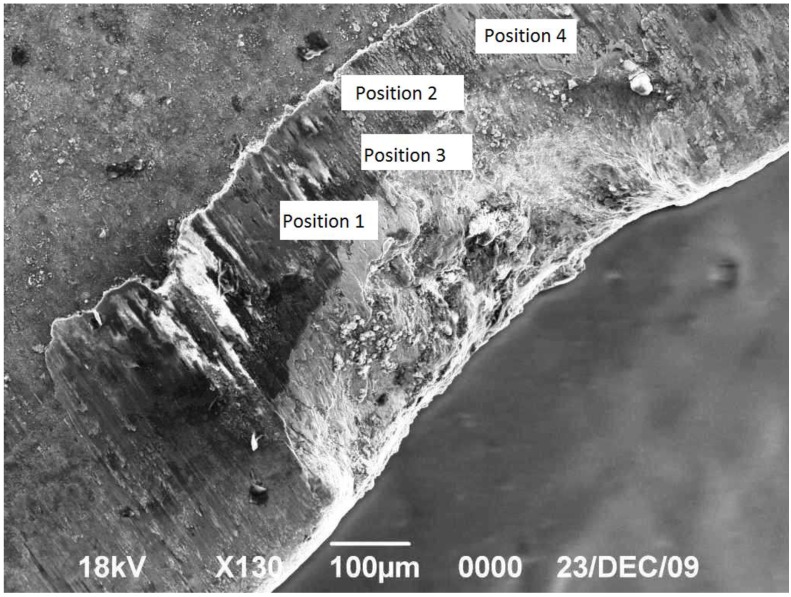
Scanning electron microscopy (SEM) micrograph of the notch wear of one of the coated carbide inserts used in this study.

**Figure 2:**
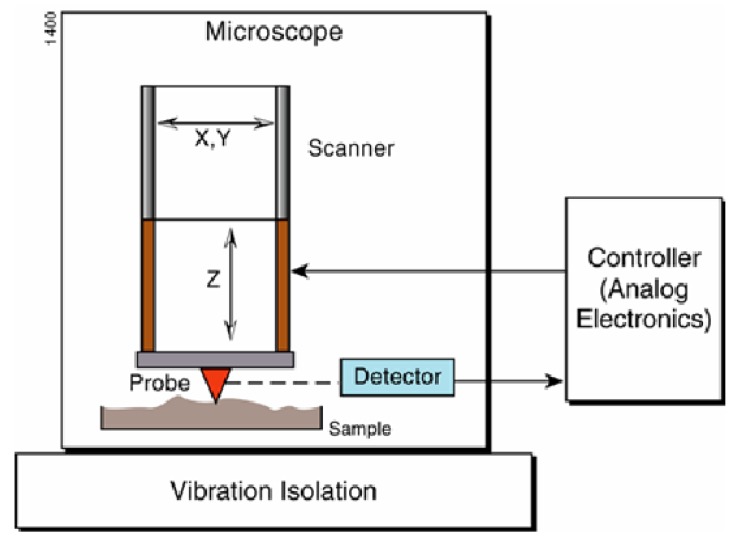
Basic tip movements in atomic force microscopy (AFM) [14].

Different techniques can be applied to assess the integrity of the coating after manufacturing such as: the nano-indentation test to assess the mechanical properties of thin coatings, the scratch techniques to determine the adhesion strength and load bearing capacity, the interfacial fatigue testing to measure the cyclic bond strength of the coating under dynamic loads, the wedge impression test to measure interface toughness between films and substrates using numerical methods, and the tensile cracking approach to evaluate both the cohesive strength of the coating and the interfacial adhesion strength between the coating and the substrate [10,11]. Assessment of the integrity can be achieved using different microscopic techniques such as optical microscope (OM), scanning electron microscopy (SEM) and/or atomic force microscopy/scanning probe microscopy (AFM/SPM). Optical microscopy provides only limited information about the surface morphology and SEM is usually used for macro-scale examination of the surface topography and fractography. For example, the SEM micrograph in Figure 1 introduces a clear and global vision about the gross wear and failure of the cutting edge. Edge deformation spreads over a relatively wide area and it is so severe that its depth is extended through the three coating layers reaching the insert substrate. Data about this specimen will be used later in the current study as AFM has been used to scan the deformed area at four different locations as marked on the figure. In such situations, AFM techniques have a high potential for integrity assessment at the micro and nano-scale of the surface. The atomic force microscope (AFM), which was invented in 1986 by Binnig, Quate, and Gerber [12], has become an indispensable tool for investigators in many fields applications; physical, chemical, tribologyical and mechanical materials properties, biological sciences and, biomechanical and electromechanical. AFM uses a mechanical probe with an ultra small tip to scan a surface sample in both X and Y directions and to sense the corresponding vertical height Z, thereby generating a magnified, or three-dimensional images of surfaces down to nanometer resolution, Figure 2. A feedback control system responds to those changes by adjusting the tip-sample distance in order to maintain a constant deflection/ distance to the sample surface [13]. It is essentially this vertical movement of the tip that translates into a topographical image of the surface with accuracy of few µm or less. The main aim of this paper is to study and discuss the integrity of coated and uncoated carbide inserts using AFM analytical parameters. This is to determine the feasibility of using AFM features to establish an efficient firm and time saving testing routine for use by tool researchers, designers, developers and quality controllers to improve the characteristics of the manufactured inserts or to develop new advanced types.

## 2. Experimental Setup and Hardware Setting

**Figure 3:**
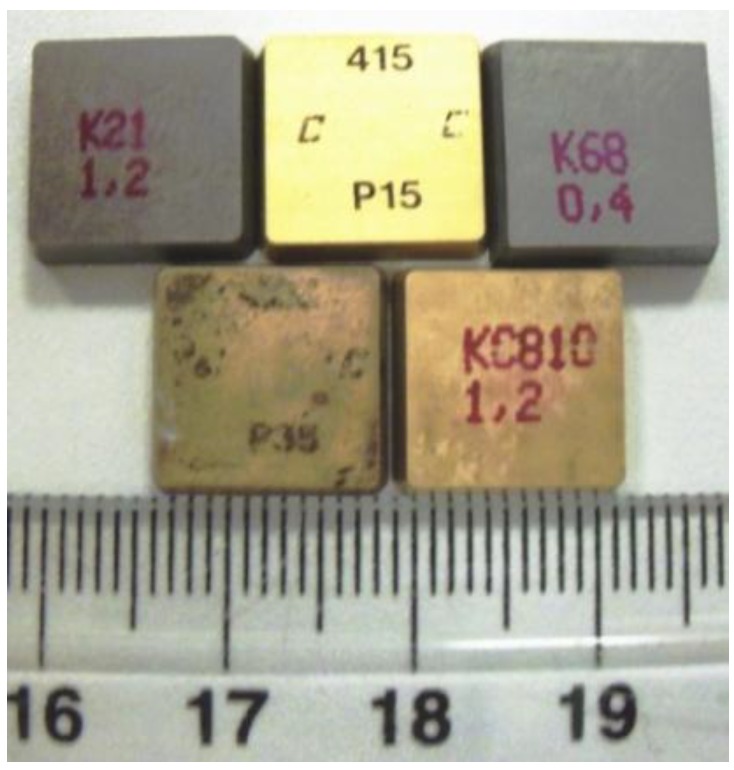
Types of employed coated and uncoated carbide inserts (cm scale with mm subunits).

Five as received inserts types were tested throughout the different stages of this study; two uncoated and three multi-layer coated with a cemented carbide substrate, Figure 3. Technical specification of the five types of inserts used in this study is listed in detail in Table 1. Inserts are of SPUN 12 03 12 configuration (thickness = 3.18 mm, r = 1.2 mm and l = 12.7 mm, clearance angle = 5–7 rake angle = 6). To avoid possible testing scratches from AFM probe, a new sample was employed for each scan run.

The NanoScope IV MultiMode Atomic Force Microscope (AFM) Scanning Probe Microscope (SPM) in contact mode was used in this study. The contact mode was selected to suit the tribological nature and the object of the current study. The NanoScope™ software [14] was used as a digital control of the AFM processes. Software features allowed for all operations including preparation and manipulation of the microscope before, during and after scanning (offline analysis procedures). To ensure system stability during scanning, a scan rate was set to 1.5–2.5 Hz. Scanner of the type AS-12 (E), with a max scan size of (12 × 12 μm) and a vertical range up to 2.5 μm, was used throughout the entire study.

**Table 1:** The specification of the five coated and uncoated inserts used in this study.

Insert type	ISO Application Range	Feature	Applications
Kennametal K68	M10-20 K05-20 (ANSI Range: C3)	low binder content, unalloyed grade WC/Co fine-grained grade	excellent abrasion resistance for machining cast irons, austenitic stainless steels, non-ferrous metals, nonmetals
Kennametal K21	M10-20 K05-20 (ANSI Range: C3)	low binder content, unalloyed grade WC/Co fine-grained grade	excellent abrasion resistance for machining cast irons, austenitic stainless steels, non-ferrous metals, nonmetals
Kennametal multicoated KC810 CVD coated carbide	M10-20 K05-20 (ANSI Range: C3)	1 μm TiN–3 μm A_l2_O_3_—5 μm TiC	general steel machining at low to moderate speeds
Sandvik CVD multicoated GC415	(P05-30, K05-20, C6-8)	1 μm TiN–3 μm Al_2_O_3_—5 μm TiC	turning steel and cast iron
Sandvik CVD multicoated GC435	ISO P35 range	1 μm TiN–3 μm Al_2_O_3_—5 μm TiC	steel cutting with decreasing rates of plastic deformation and growth of thermal and mechanical fatigue cracks

## 3. Results and Discussion

### 3.1. Selection of Data Types (Captured Image)

Available data types in Atomic Force Microscope (AFM) are: Height, Deflection and Friction. In general, while Height and Deflection data provide information about the surface topography along the scan axis, Friction image produces information about the lateral movement of the cantilever perpendicular to the scan direction.

**Figure 4:**
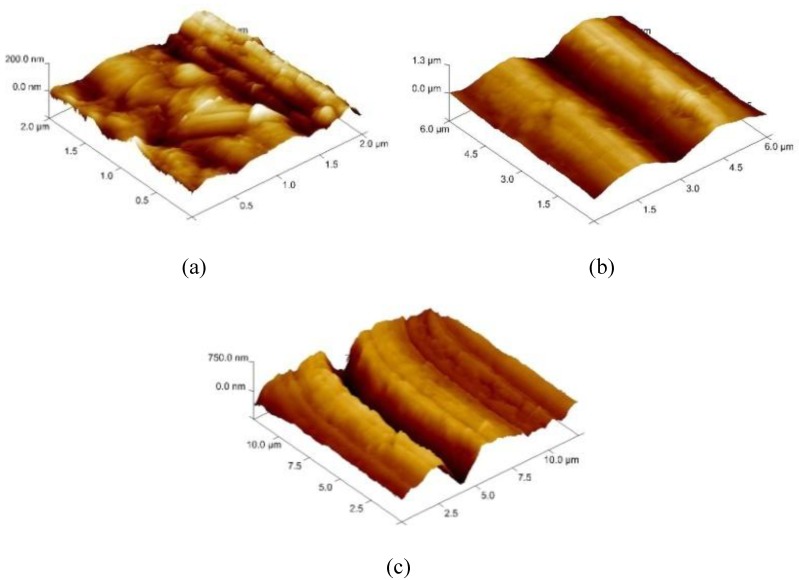
Three dimensional Height images for different scan sizes for K68 uncoated carbide inserts of **(a)** 2 μm; **(b)** 6 μm and **(c)** 12 μm.

**Figure 5:**
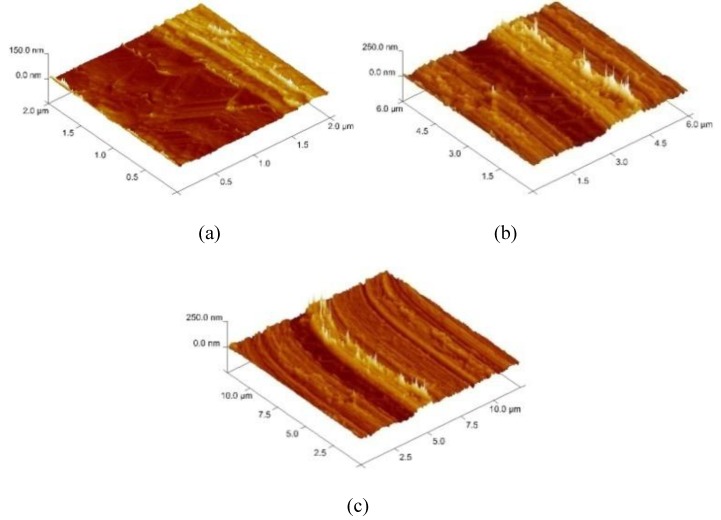
Three dimensional Deflection images for different scan sizes for K68 uncoated carbide inserts of **(a)** 2 μm; **(b)** 6 μm and **(c)** 12 μm.

**Figure 6:**
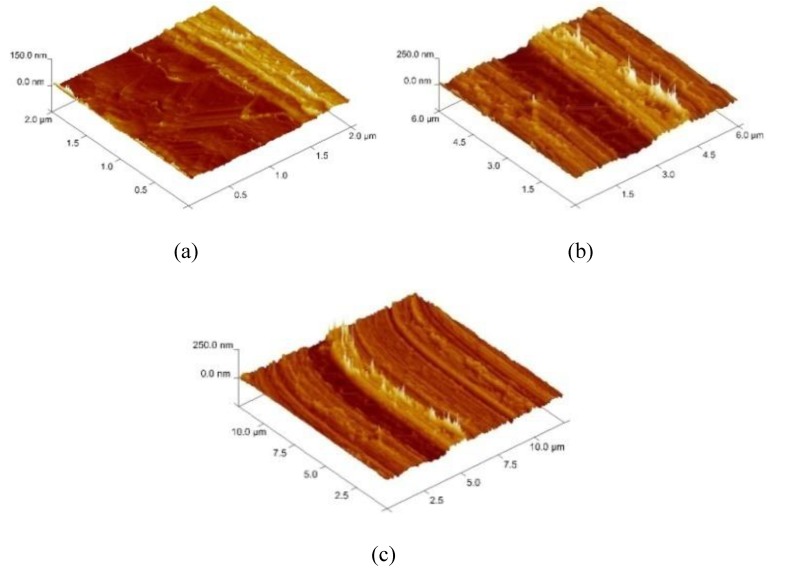
Three dimensional Friction images for different scan sizes for K68 uncoated carbide inserts of **(a)** 2 μm; **(b)** 6 μm and **(c)** 12 μm.

**Figure 7:**
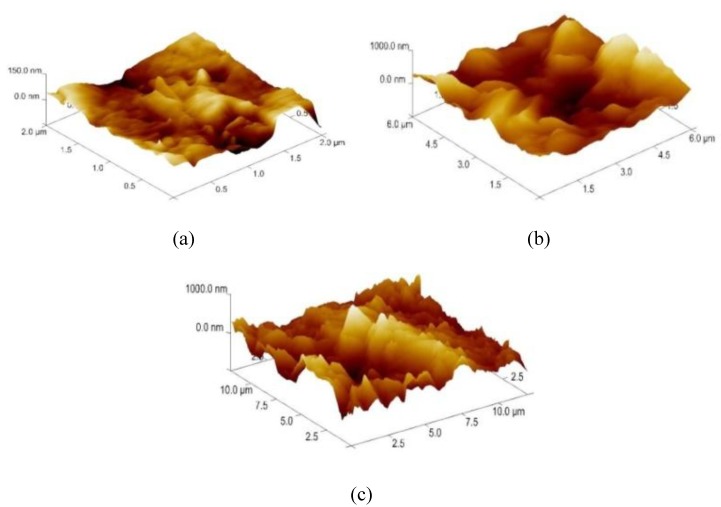
Three dimensional Height images for different scan sizes for GC435 coated carbide inserts of **(a)** 2 μm; **(b)** 6 μm and **(c)** 12 μm.

**Figure 8:**
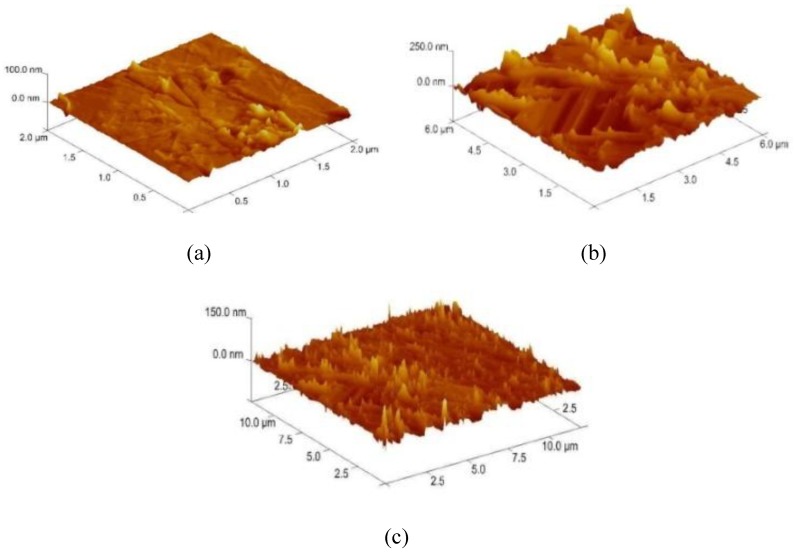
Three dimensional Deflection images for different scan sizes for GC435 coated carbide inserts of **(a)** 2 μm; **(b)** 6 μm and **(c)** 12 μm.

**Figure 9:**
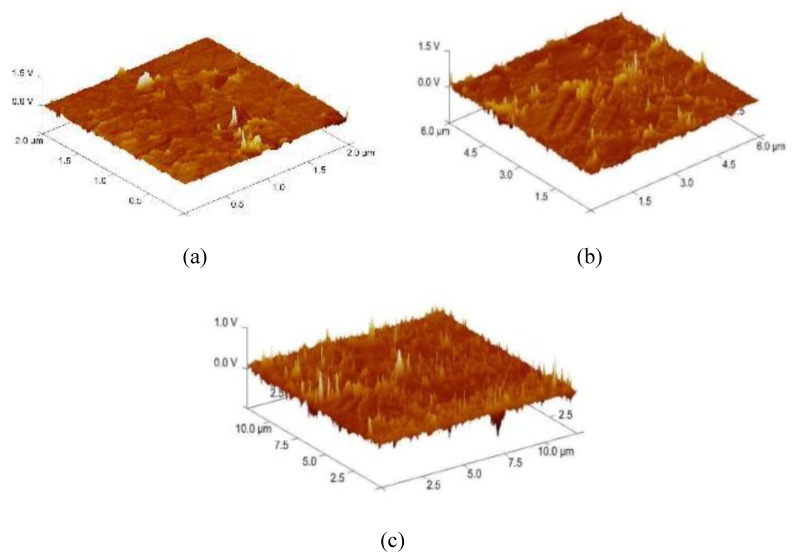
Three dimensional Friction images for different scan sizes for GC435 coated carbide inserts of **(a)** 2 μm; **(b)** 6 μm and **(c)** 12 μm.

**Figure 10:**
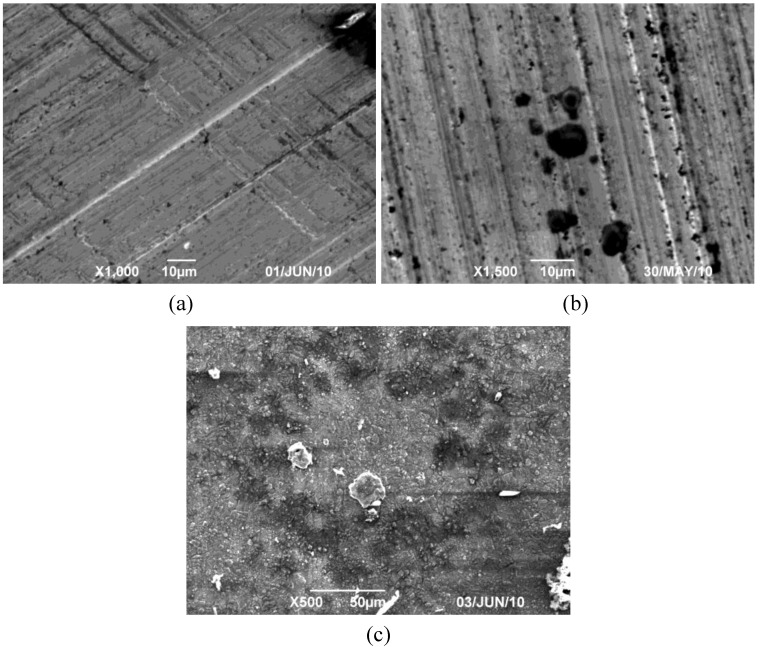
SEM surface micrograph showing defects of the uncoated and multilayers coated carbide inserts. **(a)** surface groove (K68, sample 2); **(b)** high surface roughness (K68, sample 4); **(c)** droplets of coating spallation and delamination (GC415, sample 2).

In order to select the appropriate data type, individual scans were carried out for the coated and uncoated inserts, with the three different data types images (Height, Deflection and Friction) being simultaneously captured in both the two and three dimension views. Data were coded and stored for subsequent offline analysis. The procedures were repeated considering three different scan sizes: low (2 μm), medium (6 μm) and maximum available (12 μm). Samples of the captured data (images) are shown in Figure 4, Figure 5 and Figure 6 for K68 uncoated and, in Figure 7, Figure 8 and Figure 9, for GC435 coated inserts. Each graph shows a three-dimensional view where the X-Y plane represents the scanned area while the response (Height, Deflection or Friction) level is represented by the Z axis. The sample displayed in Figure 4, Figure 5 and Figure 6 is of K68 uncoated carbide type, Table 1. A surface flaw, Figure 10a, in a form of a recess or surface groove was detected. This surface imperfection is basically due to some improper manufacturing or preparation and finishing (grinding) procedures. The depth of the groove is detected by the analysis, sample 2, Table 2, to be as deep as 810 nm. Such defects may degrade the insert surface finish and, consequently affects its performance whether it is used as a plain (uncoated) insert or is prepared for further coating process. Also, Figure 7, Figure 8 and Figure 9 explain similar micrographs to explain the possible existence of defects in the coating layers. Figure 10 shows the SEM micrographs of many forms of the observed surface defects such as: droplet, spallation, delamination and macroparticles of the coating layer. Figure 10c shows a SEM micrograph for GC415 coated inset, sample 2, Table 3, where frequent droplets were observed on the surface of the insert. The droplet configuration is better visualized and further analyzed using the appropriate combination of data type and scan size.

The images indicate the existence of mutual interaction between Deflection and corresponding Friction images as tip of the probe usually exhibits tilting laterally when it moves along the scanning direction. Generally, it can be concluded that the Height data type produces an absolute judgment of the surface roughness over the entire area along the scanned direction while Friction data introduces an attractive indication about the roughness pattern in the lateral direction. Practically, it can be stated that the captured Height data is preferable for surfaces that exhibit regularly distributed fingerprint topography with less waviness and disturbances. This is supported in what was recommended by [14], that in most instances, Height data type usually ensures an accurate topographical view.

### 3.2. Determination of the Appropriate Scan Size

To determine the most informative captured data, each specimen was independently scanned using three different scan sizes; 2, 6 and 12 μm (as a limiting AFM capacity), Figure 4, Figure 5, Figure 6, Figure 7, Figure 8 and Figure 9. As shown in Figure 4, the extent of the surface defect (recess, Figure 10a) was precisely described by the widest scan size of 12 μm. Some features would be lost when smaller scan sizes, Figure 4a, and b, were considered. The same conclusion was reached regarding the existence of the droplet defect on the surface of sample 2, Table 3. A full configuration of the droplet flaw was clearly described using 12 μm scan size in comparison to what was obtained by smaller scan sizes or by SEM micrograph, Figure 10a. Generally, the use of a wider scan size usually produces more common, integrated and more informative view of the surface topography. This is usually accompanied with adequate details permitting the proper examination and the detection of the relatively wide surface defects and its complete waviness measure [15,16]. Therefore, as a general rule, it is better to start an investigation with the widest scan size available. In situations, however, a smaller scan size can be useful to provide some nanoscale characteristics of the intended surface.

### 3.3. Data Analysis Techniques

A wide variety of analysis functions are available from the “Analyze” menu in the off-line mode of Nanoscope software [14] and, in the current study, only parameters relevant to surface tribological aspects are extracted and elaborated. Investigated parameters include the “Roughness” command and the informative “Section” command.

#### 3.3.1. “Roughness” Analysis Parameter of Surface Topography

The “Roughness” parameter generates a wide variety of statistics of the surface topographical aspects including classical roughness values, peak and summit texture data and surface area calculations for the entire image. Among the many parameters available in the “Roughness” analysis, four relevant measures are selected to analyze the captured data. The four measures are the “Average roughness” Ra, the “Image RMS” (Rq), the “Image area difference %” and the image “Z-range”. Analysis and results considering the above mentioned four basic roughness parameters accompanied by their associated qualitative three-dimensional thumbnails are listed in Table 2 and Table 3. Table 2 lists the values of the four measured roughness parameters for K68 and K21 uncoated inserts. Among the four scanned samples of K68 uncoated carbides (Samples 1–4), all parameters values support the idea that sample 3 is a normal defect-free specimen and it can be considered as a reference for the rest of the samples. A SEM micrograph of sample 4 is shown in Figure 10b where high rough disturbances dominated the entire surface of the insert. In comparison to the counterpart values for the normal sample 3, all parameters are of much greater levels reaching 350%, 2,300%, 157% and 146% increase for Z-range, area difference %, Rq and Ra, respectively. For sample 2, Table 3, that is shown by the SEM micrograph, Figure 10a, corresponding values were about 76%, 20%, 128% and 119%. These values, in comparison to those for sample 4, indicate that, in some situations where localized defects dominate, sample 2, relative assessment is not accurate enough and more analysis using the most appropriate and specific parameter is required. This remark is supported by the wide variation in the values of roughness measures of K21 uncoated carbides, samples 1–2, Table 2. Whereas, for sample 2, a localized surface defect exists, the surface topography is better represented either by Ra or Rq.

Table 3 lists values of the roughness parameters for the coated GC415 inserts including four intact samples (samples 1–4) in addition to one worn insert (sample 5). For the same worn specimen sample 5, four scans were performed on some preselected locations defined by positions 1 to 4 as shown in Figure 1. When the values of each of the four roughness parameters of such a sample were compared to the corresponding reference values, as the average mean of the measures of samples 1–4, the percent increases were found to be; 1,400%, 18.6%, 202% and 154% for Z-range, area diff.%, Rq and Ra, respectively. As shown in Figure 1, wear mode was of rubbing nature giving a misleading indication of surface roughness improvement. However, for positions 2 and 3 (sample 5, Table 3), the wear mode is of an irregular nature that is interpreted as increasing the levels of all the roughness measures considered. This limits the feasibility of using AFM analysis for the worn specimen and, the use of a different examination technique such as SEM is preferred.

#### 3.3.2. “Section” Analysis of Surface Topography

**Figure 11:**
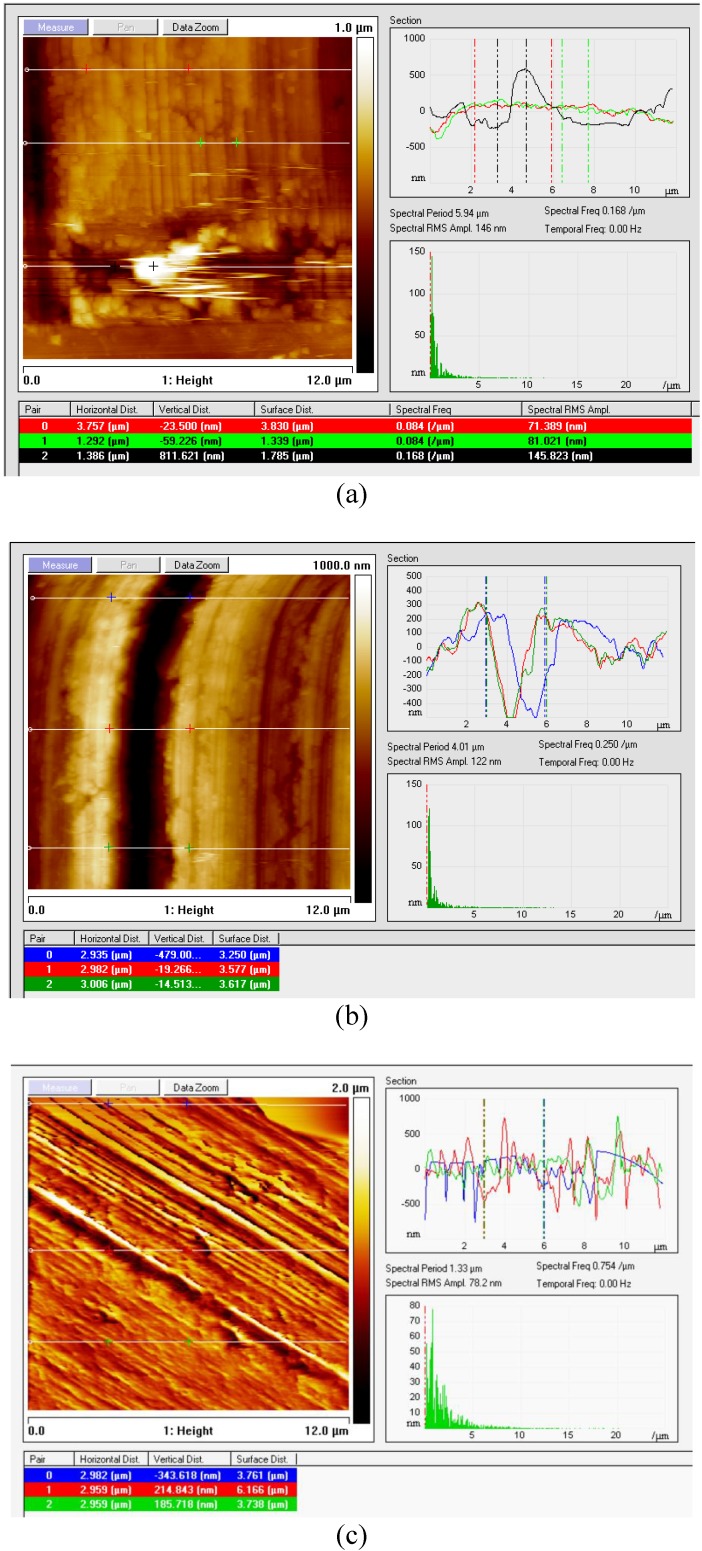
Section surface analysis of K68 uncoated carbide inserts **(a)** Multi horizontal sections for Sample 1 (partially defected); **(b)** Multi horizontal sections for Sample 2 (cracked surface) and **(c)** Multi horizontal sections for Sample 4 (overall rough surface).

The “Section” feature in NanoScope software offers a useful tool to quantitatively investigate the topography of the surface localized defects [14]. Figure 11 shows the use of the “Section” command for the K68 uncoated carbide samples 1–3, Table 2, using the “Height” two-dimensional image with 12 μm scan size. Three horizontal reference lines were allocated to provide information about the surface topography over different locations of the scanned area. More features than those provided by “Roughness” parameters were obtained including the width and the frequency of the existing flaws. For instance, in Figure 11b, the groove width was determined to be of 3 μm regular width.

As shown in Figure 11c, data for the defective sample 4 reveals a height range of 1.5 μm comparing to 0.5 μm for the reference sample 3, Table 2.

**Figure 12:**
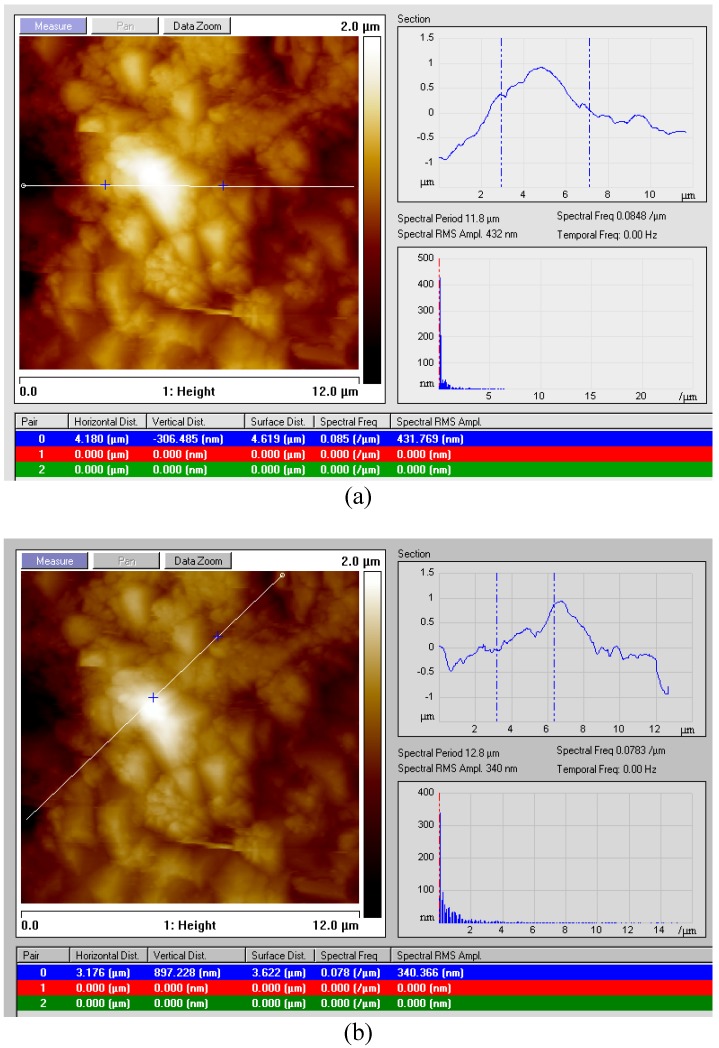
Section surface analysis of GC415 coated carbide inserts indicating the configuration of the coating droplet.

In order to get more information about the configuration of the droplet defect on the surface of the GC415 coated sample 2, Table 3, both horizontal and diagonal sectioning reference lines were allocated, Figure 12. It is shown that the droplet is of approximate elliptical shape with about 3 and 6 μm diagonals. Also, graphs indicate that within the scanned area, there is only one droplet occurrence on the scanned area (unity frequency).

As shown in Figure 11 and Figure 12, the accompanied table at the bottom of each graph provides some useful information about the marked surface area being examined.

**Table 2:** Recommended surface controlling parameters for uncoated carbide inserts.

Sample No.	Roughness data				Section Data		Notes & Remarks
	Zrange (nm)	Area Diff.%	Rq (nm)	Ra (nm)	Spectral RMS (nm)	Height Range (nm)	
Sample 1-K68	1,281	36.1	119	83.9	146	−250 To 600	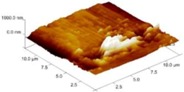
Sample 2-K68	1,114	11.1	192	144	12	−500 To 310	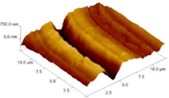
Sample 3-K68	634	9.24	84.3	65.8	101	−300 To 200	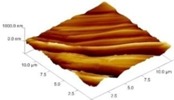
Sample 4 = K68 (defect)	2,855	222	217	161	782	−750 To 750	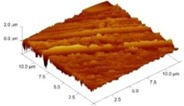
Sample 5 = K21	674	8.3	54.2	35.8	52.9	−140 To 215	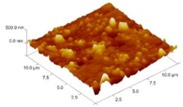
Sample 6-K21 (Defect)	1,951	16.7	398	341	449	−1,000 To 700	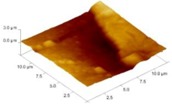

**Table 3:** Recommended surface controlling parameters for GC415 multilayer coated carbide.

Sample No.	Roughness data				Section Data		Notes & Remarks
	Zrange (nm)	Area Diff. %	Rq (nm)	Ra (nm)	Spectral RMS (nm)	Height Range (nm)	
Sample 1	1,224	22.3	186	112	208	−500 To 485	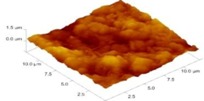
Sample 2	1,954	21.4	291	227	432	±1,000	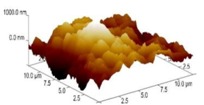
Sample 3	1,355	12.4	168	130	86.7	−350 To 390	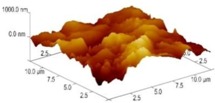
Sample 4	1,148	18.3	164	128	146	−450 To 490	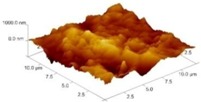
Sample 5 (worn-Pos. 1, Figure 1)	612	5.84	90.2	73.4	78.9	−160 To 200	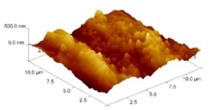
Sample 5 (worn-Pos. 2, Figure 1)	2,912	107	500	398	818	−800 To 2,000	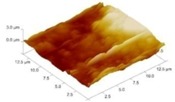
Sample 5 (worn-Pos. 3, Figure 1)	2,462	31.1	311	236	815	0 To 2,000	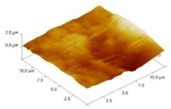
Sample 5 (worn-Pos. 4, Figure 1)	978	8.09	123	97.4	160	±250	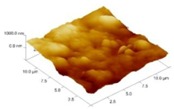

### 3.4. A Recommended Strategy for Prior Surface Integrity Assessment

Depending on the results obtained in this study, the use of AFM/SPM features for prior examination and assessment of surface topography of coated and uncoated cutting inserts is justified especially when used with other microscopy techniques such as SEM. The ability to deal with and to handle the technique, however, depends to great extent on many technical and economical judgments. Therefore, an optimization strategy for problem manipulation is proposed in Table 4 with recommended information about hardware and software settings in addition to the selection of the appropriate data analysis parameters. Parameters are optimized based on the type of the detected surface disturbances. In the majority of situations many analysis features are concomitantly provided and the preference among them usually requires user technical intervention. Independent technical opinions should be based on the functional objectives set by designer and the decision makers. However, many technical and practical relevant issues were raised throughout the experimental and analysis stages and these are discussed in the following paragraphs.

As a general practical rule, the Z-range and Area Diff. % measures in “Roughness” parameter usually should be started in the first examination instant. These offer a good prior general indication about surface topography and its quality. Whenever a regular roughness pattern is observed, with a “spread out” nature all over the entire surface, many “roughness” measures can be beneficial to determine the general characteristics of the topography. When manufacturing imperfections are observed, the height range in the “Section” should be of choice.

However, when worn surfaces are examined using AFM/SPM, the surfaces are always deformed in various rubbing, hardening and chipping modes [17,18,19]. In such situations, surface topography tends to have longer wavelength with small random asperities and, in general, roughness analysis may offer little relevant information. Further examination procedures using SEM or even high sensitivity light microscopy can be more feasible. Nevertheless, it is found that the “Height” range in “Section” can provide some useful information regarding the severity and the nature of the developed wear mode.

Although, in the current study, several types of surface defects and flaw of coated and uncoated carbide inserts were detectable, the criticality of these defects and flaws are not apparent and cannot be claimed. Therefore, it is beneficial especially with the development of the AFM to assess the integrity of coatings by finding out the failure initiation factors for the coating layers and the role of these surface defects. Such studies can provide benchmarks for the different types of failure mechanisms and provide an important insight for quality assurance of coating.

**Table 4:** The specification of the five coated and uncoated inserts used in this study.

Seq.	Type of surface Imperfections	Roughness data				Section Data		Hardware and Software Setting
		Zrange (nm)	Area Diff. %	Rq (nm)	Ra (nm)	Sp. RMS (nm)	Height Range (nm)	
1	Preliminary surface examination	•	•		•			a) Use at least three different samples. b) Use maximum available scan size. c) Use “Height” image.
2	Normal defects free surface	•	•		•		•	
3	Regular widespread roughness pattern	•	•		•		•	
4	Tiny localized surface defects	•	•				•	a) Compare with normal reference topography (Proced. 2).
5	Manufacturing coating imperfections	•					•	
6	Worn edge	•					•	a) Further SEM or/and OM is preffered.

## 4. Conclusions

Prior examination of the coated and uncoated cutting inserts can contribute to the resolution of tool wear and life variability problems especially if they are used in fully automated machining systems. The main objective of the current study was to come up with an offline feasible strategy using AFM/SPM facilities to assess the surface integrity of coated and uncoated cutting tool’s carbide inserts prior to use and probably within an efficient quality control strategy by the manufacturers and developers.

Among the wide spectrum of information offered by the various features of AFM/SPM in contact mode, only relevant measures were extracted and utilized. The “Height” imaging, in association with the maximum available scan size, was selected and found to be sufficiently good enough to capture most types of surface imperfections and to produce accurate information about the topography of the intended surface.

Among analyses features available in the system software, “Roughness”, and “Section” parameters were found appropriate to analyze the surface topography and its relevant tribological characteristics. Based on the results obtained, an optimized examination and manipulation strategy has been proposed. This presents effective, direct and time saving procedures to guide designers, developer, and quality controllers to deal with the issue.

However, when used (worn out) surfaces are examined using AFM, the surfaces are always deformed in various rubbing, hardening and chipping modes. In such situations, surface topography tends to have longer wavelength with small random asperities and, in general, roughness may offer little relevant information. Further examination procedures using SEM or even high sensitivity light microscopy can be more feasible.
